# Do I trust you when you smile? Effects of sex and emotional expression on facial trustworthiness appraisal

**DOI:** 10.1371/journal.pone.0243230

**Published:** 2020-12-03

**Authors:** Dina F. Galinsky, Ezgi Erol, Konstantina Atanasova, Martin Bohus, Annegret Krause-Utz, Stefanie Lis

**Affiliations:** 1 Institute of Psychiatric and Psychosomatic Psychotherapy, Central Institute of Mental Health, Mannheim, Germany; 2 Medical Faculty, Heidelberg University, Heidelberg, Germany; 3 Institute of Psychology, Leiden University, Leiden, Netherlands; University Hospitals Tubingen: Universitatsklinikum Tubingen, GERMANY

## Abstract

**Background:**

Trust is a prerequisite for successful social relations. People tend to form a first impression of people‘s trustworthiness based on their facial appearance. The sex of the judging person and its congruency with the sex of the judged people influence these appraisals. Moreover, trustworthiness and happiness share some facial features, which has led to studies investigating the interplay between both social judgments. Studies revealed high correlation in judging happiness and trustworthiness across different facial identities. However, studies are missing that investigate whether this relationship exists on a within-subject level and whether in-group biases such as the congruency between the sex of the judging and judged individual influence this relationship. In the present study, we addressed these questions.

**Methods:**

Data were collected in an online-survey in two separate samples (N = 30, German sample, N = 107 Dutch sample). Subjects assessed the intensity of happiness and trustworthiness expressed in neutral and calm facial expression of the same characters (50% males, 50% females). Statistical analyses comprised rm-Anova designs based on rating scores and estimates of within-subject associations between both judgments.

**Results:**

Our findings replicate high correlations between happiness and trustworthiness ratings across facial identities based on average scores across participants. However, the strength of this association was strongly dependent on the methodological approach and inter-subject variability was high. Our data revealed an in-group advantage for trustworthiness in women. Moreover, the faces’ sex and emotional expressions differentially influenced the within-subject correlation between both judgments in men and women.

**Conclusion:**

Our findings replicate previous studies on the association between happiness and trustworthiness judgments. We extend our understanding of the link between both social judgments by uncovering that within-subject variability is high and influenced by sex and the availability and appraisal of positive emotional facial cues.

## Introduction

From an evolutionary perspective, being part of a social group is essential for survival [1,2]. Prerequisite for experiencing a sense of belonging and building affiliations is that people trust each other [3]. Trust is developed in different ways [4]: learning about cooperative and fair behaviours of others enhances trust. Similarly, people may develop trust in a person based on a given context, e.g. through descriptions of a person’s attributes provided by others [5]. However, even if people face a stranger for the first time, they tend to form an impression of the other’s trustworthiness. Studies suggest that people do so in a very consistent way: inter-observer agreement is high in trustworthiness inferences based on facial appearance and this is observable already during childhood [6–8]. There is little evidence that these inferences accurately reflect the personality of a person suggesting that these social judgments reflect prejudices and are of limited usefulness as a basis for decisions in everyday life [9]. Nevertheless, first impressions of trustworthiness have important consequences: trustworthiness judgments influence future social interactions and also predict important social outcomes, such as electoral success and sentencing decisions [10]. This emphasizes the importance to understand which factors modulate these judgments. Factors that influence the variability of trustworthiness appraisals of strangers comprise membership in the same social group for the judging and judged individuals, as well as social cues seemingly related to trait or state attributes of an individual.

Similarity between the judging and judged person is an important factor for trust. Across different sources of similarity such as university membership, nationality or country of residence, people are likely to place more trust in in-group members than in out-group members [11,12]. One feature suited to define group membership at first impression is sex. Although several studies investigated the relation between sex and trust, their results are inconsistent. Most studies suggest a higher trustworthiness of female faces compared with male faces with small to medium effect sizes [13,14]. However, only some of these studies suggest that this effect is specific for women, i.e. that it is due to an in-group bias [13]. Moreover, findings from Dzhelyova and colleagues [15] even suggest the existence of a sex stereotyped association of women with trustworthiness and men with untrustworthiness: trustworthy female faces and untrustworthy male faces were classified faster and more accurately in regard to their trustworthiness by both men and women.

However, biases due to sex or in-group features of an individual alone cannot explain the high inter-observer agreement of first impression trustworthiness. This initiated studies that investigated whether trustworthiness is determined by structural and dynamic features of a face. Structural facial features such as higher inner eyebrows, pronounced cheekbones, wide chins and shallow nose sellions are associated with higher trustworthiness judgments [16]. Beyond such physiognomic features, the emotional expression of a face is a crucial factor influencing assessments of trustworthiness. Particularly, the expression of happiness seems to be a social cue associated with trustworthiness. This relation was found in studies presenting prototypical facial expressions with a medium to high intensity of happiness [17–21], as well as in those that investigated the effects of subtle expressions of happiness in neutral faces [7,22–24]. A variation of facial features along the dimension of trustworthiness and happiness revealed that increasing the intensity of happiness resulted in higher trustworthiness ratings [21]. Vice versa, increasing trustworthiness resulted in ratings of a higher intensity of happiness, while increasing untrustworthiness attenuated intensity ratings of happiness [19]. In line, variability in trustworthiness judgments between different images of the same identity is high and may even exceed variability between different identities [25].

In sum, several studies consistently support the role of perceptual similarity between trustworthiness and the emotional expression of happiness [19,21]. However, so far, trustworthiness and happiness were judged most often by different groups of participants [e.g. 25,26]. In consequence, the covariation between social judgments was calculated as correlation of mean happiness and mean trustworthiness ratings based on averaging ratings across participants separately for different facial stimuli. This informs us about how consistently happiness and trustworthiness vary across a range of facial identities. However, information is widely missing on whether people differ in how the evaluation of a positive emotional expression influences first impression trustworthiness judgments and whether this association is influenced by an individual’s sex and its congruency with the sex of the judged face.

To fill this gap, we investigated the variability between individuals concerning the interplay between facial emotion evaluations and trustworthiness impressions within a single subject. In contrast to most previous studies, we manipulated the emotional expression of the facial stimuli and asked the same participants to assess happiness and trustworthiness in both neutral and calm facial expressions of the same male and female characters. In general, we expected that 1) low intense positive facial expressions result in higher trustworthiness and happiness ratings than neutral expressions, that 2) female faces are judged as more trustworthy than male faces and that 3) this effect is particularly pronounced in women due to an in-group bias. In regard to the between-judgment covariation, we expected in line with previous studies that 4) there is a high positive correlation between experienced happiness and trustworthiness in both neutral and calm faces when this relation is calculated based on the ratings for the single facial stimuli averaged across participants. Moreover, we analysed the relation between both types of social judgments separately for each participant. We were interested in 5) whether people differ in the strength of this association depending on the experimental factors and the participant’s sex. Finally, we explored whether participants’ features such as their concerns about justice, as well as their general health and current mood influence differences in between-judgment associations between participants.

## Methods

### Participants

We measured social judgments in two independent samples in two anonymous online-surveys.

Sample 1 was recruited in Germany by distribution of the survey link through the social networks of the researchers and Facebook groups of students of Mannheim University and Heidelberg University. The study was approved by the Research Ethics Board of the Medical Faculty, Heidelberg University, Germany.

Sample 2 was recruited in the Netherlands by distribution of the survey link using the Leiden University Research Participation Platform SONA. Psychology students earned 1 credit point as part of the requirement of research participation in the first year. The study was approved by the Psychology Ethics Board of the University Leiden, Netherlands.

In both samples, we assessed psychological distress with the Brief Symptoms Inventory-18 (BSI-18, [27], German version [28]. The BSI-18 measures depression, anxiety and somatization with 6 items using a 5-point Likert scale. Scores range from 0–24 for the three subscales and 0–72 for the total score. Current emotion state was assessed with the arousal and valence scales of the Self-Assessment Manikin before the start of the task (9-point Likert scale, range 0–8, SAM; [29]. Additionally, we used the short version of the Justice Sensitivity Inventory to assess participants’ concern about justice from the perspective of a victim, an observer, a beneficiary and perpetrator [30]. The different perspectives are assessed with the composite score of 2 items each, assessed on a 6-point Likert scale. Higher scores indicate stronger concerns about justice (range 1–6). For sample descriptions, see Table 1.

**Table 1:** Samples’ description.

	Sample 1	Sample2
**N**	30	106
**Sex (m/f)**	7/23	25/81
**Age**	31.8±11.7	20.8±4.5
**Education Abi/RS/other/r.a**.	28/2/0/0	79/18/6/3
**Nationality German/Dutch/other**	25/0/5	0/102/4
**Partner Status Single/With partner/r.a**.	8/20/2	65/39/2
**SAM-valence**	3.6±1.5	3.7±1.5
**SAM-arousal**	5.9±1.8	6.2±1.7
**BSI-total**	27.6±6.1	31.1±11.0
**BSI-somatic**	8.2±2.0	8.9±3.2
**BSI-depression**	9.8±4.5	11.3±4.7
**BSI-anxiety**	9.6±2.6	10.9±4.7
**JS-victim**	3.3±1.3	3.9±1.0
**JS-observer**	4.9±1.1	3.6±0.9
**JS-beneficiary**	3.6±1.4	3.4±1.0
**JS-perpetrator**	5.9±1.2	4.1±1.1

*From the total of 107 participants in sample 2, one female participant was excluded since she reported to have done the survey not on her own. r.a. = refused to provide this information.

### Experimental procedure

During the experiment, participants judged facial stimuli. We experimentally manipulated the type of the social judgment task, as well as the emotional expression and the sex of the presented facial stimuli. Participants assessed how strongly the presented face expressed happiness and trustworthiness (independent variable: ‘task’). They indicated their response on a 7-point Likert scale ranging from 1–7 (‘not at all’ to ‘very much’). We presented 50% male and 50% female faces (independent variable: ‘sex-stim’). Each character was presented with a neutral and a calm expression (independent variable: ‘emotion’).

In total, we selected 18 male and 18 female stimulus characters from the Interdisciplinary Affective Science Laboratory Face Set (IASLab Face Set; http://www.affectivescience.org/face-set.shtml). For each of the characters, we used a neutral and calm facial expression. We chose calm faces to allow for a variation in the ratings of the intensity of the emotional expression. Calm faces have the same valence as happiness, but a lower level of arousal (see affective circumplex model [31]).

Participants rated happiness and trustworthiness in separate blocks presented in randomised order. Within each block, the 72 pictures were presented in randomised order.

At the end of the survey, participants had to indicate whether they completed the survey alone and answered honestly.

### Statistical analyses

In line with previous studies, we analysed first whether the type of the social judgment and the emotional expressions of the facial stimulus influenced the intensity of an expression ascribed to a face. This 2x2 rm-ANOVA-design with the repeated measurement factors ‘emotional-expression’ (‘emotion’: calm/neutral) and type of social judgment (‘task’: happiness/trustworthiness) was analysed separately for both samples. Testing for normality of the residuals (Kolmogorov-Smirnov, visual inspection of Q-Q Plots) revealed that the assumption of a parametric analysis was violated in some cells of the design. In line, we applied a non-parametric approach using a rank-aligned ANOVA [32]. In a second step, we extended this design by the independent factor ‘sex’ and the repeated measurement factor formed by the sex of the presented faces (‘sex-stim’) resulting in a 2x2x2x2 design. For this analysis, we combined the two samples recruited in Germany and the Netherlands, to increase the number of cases for men since women were overrepresented in both samples. The Levene’s test based on medians indicated no violation of the assumption of variance homogeneity. Since however, the assumption of normality of the residuals was violated in some cells of the design, we applied a non-parametric rank-aligned ANOVA as for the first two analyses. Finally, we analysed the relation between happiness and trustworthiness judgments by correlation analyses (Spearman’s rho, rs) from two different perspectives. In line with previous studies, we analysed the correlations of happiness and trustworthiness judgments between different facial expressions based on rating scores per facial stimulus averaged across participants. We extended these analyses by investigating the between-subject and within-subject correlations of happiness and trustworthiness judgments. For between-subject correlations, we analysed the correlations of happiness and trustworthiness judgments between different participants based on rating scores per participant averaged across the facial stimuli. For within-subject correlations, we calculated separately for each participant Spearman correlation coefficients between happiness and trustworthiness ratings based on the ratings of the single facial stimuli. For further analyses, we transformed these coefficients to Fisher’s z-scores. By this approach, we were able to study the functional interplay between the two social judgments for each participants, i.e. to quantify to which extent a specific participant ascribed a high level of trustworthiness to those facial expressions that he/she also judged as happier. This is in contrast to investigating the covariations between mean rating scores which indicate whether participants who ascribe in general a high level of happiness to facial expressions also judge faces in general as more trustworthy. This relationship might reflect a response bias during judging social cues when for example people differ in their tendency to choose extreme response options independently of the judged facial feature. To study the effects of our experimental factors on within-subject associations between social judgments, we analysed Fisher’s z-scores in a 2x2x2 rm-ANOVA design (‘sex’ x ‘sex-stim’ x ‘emotion’). Tests for a normal distribution of the residuals (Kolmogorov-Smirnov, visual inspection of Q-Q Plots) and variance homogeneity (Levene’s test of variance homogeneity based on medians) indicated no violation of the assumptions for a parametric ANOVA. All correlations are Spearman’s rho rank correlation coefficients.

## Results

### Effects of emotional expression on social judgments

**Fig 1:**
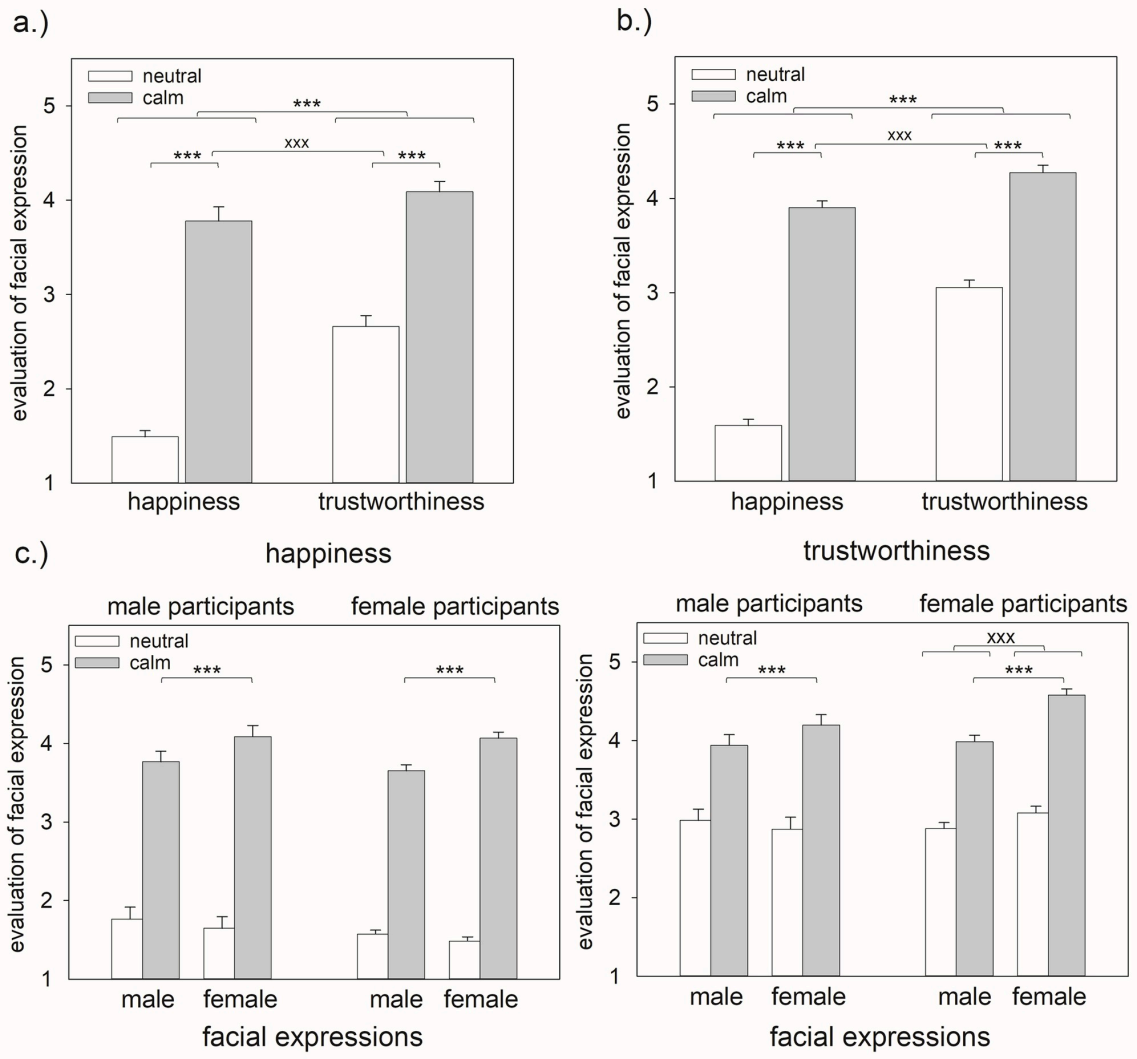
Mean and standard error for happiness and trustworthiness ratings of neutral and calm facial expressions a) sample 1, b) sample 2, c) separated for the sex of the participants and the facial stimuli (pooled for sample 1 and sample 2). Please note: Fig 1a and 1b: *** indicates p < .001 for the main effects of ‘emotion’ and ‘task’, xxx indicates p < .001 for the interaction effect; Fig 1c: *** indicates p < .001 for the interaction effects of ‘sex-stim’ * ‘emotion’, xxx indicates significant differences between pairwise comparisons with p < .001 for the interaction effect ‘sex * sex-stim * task’.

Sample 1: In general, participants rated calm faces as happier and as more trustworthy than neutral faces. Additionally, they judged faces as indicating trustworthiness to a higher extent than happiness (see main effects in Table 2, Fig 1a). Thereby, differences between happiness and trustworthiness ratings were dependent on the emotional expression, i.e. they were smaller for calm faces compared with neutral faces (see interaction effect in Table 2, Fig 1, effect size of Wilcoxon signed-rank test: neutral faces *r* = 0.86, calm faces *r* = 0.40).

**Table 2:** Results of the 2x2 robust rank-aligned-ANOVA for social judgments depending on the emotional expression (emotion) and the type of social judgment (task).

	Sample 1				Sample 2			
	df	F	p	_p_η^2^	df	F	p	_p_η^2^
emotion	1,29	301.46	< .001	.912	1,105	1016.11	< .001	.906
task	1,29	48.89	< .001	.628	1,105	172.82	< .001	.622
emotionxtask	1,29	26.36	< .001	.476	1,105	292.02	< .001	.736

Please note: Statistically significant effects are highlighted in red.

Sample 2: Analyses of sample 2 replicated the findings described for sample 1 (Table 2, Fig 1b). Participants rated calm faces as happier and as more trustworthy than neutral faces. Additionally, they judged faces as indicating trustworthiness to a higher extent than happiness (see main effects in Table 2). Similar as in sample 1, differences between happiness and trustworthiness ratings were smaller for calm faces compared with neutral faces (see interaction effect in Table 2, effect size of Wilcoxon signed-rank test: neutral faces *r* = 0.85, calm faces *r* = 0.43).

### Sex as modulating factor

Since the number of men was small in both samples, we analysed effects of the participants’ sex in a pooled sample while taking the sex of the presented faces into account.

**Fig 2:**
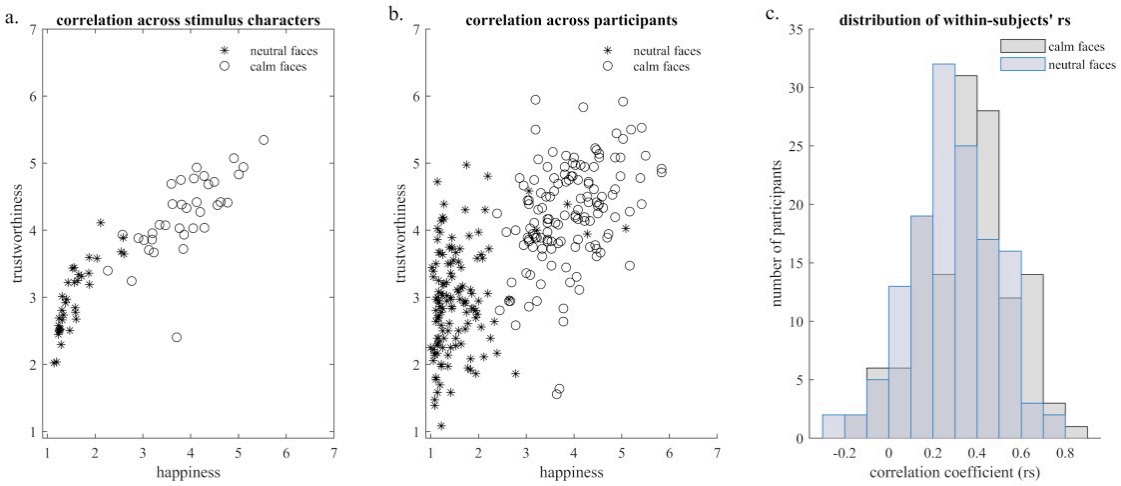
Correlation between mean happiness and trustworthiness ratings for neutral and calm faces. a) For different facial stimuli based on rating scores averaged across participants separately for each stimulus (pooled for sample 1 and sample 2). b) For different participants based on rating scores averaged across characters. c) Distributions of within-subject correlation coefficients between happiness and trustworthiness judgments.

The sex of the displayed face influenced the participants’ social judgments depending on the emotional expression (sex-stim * emotion: *F*(1,134) = 121.42, *p* < .001, _*p*_*η*^*2*^ = .556, see Table 3, Fig 1c): ratings for male and female faces differed only in calm facial expression. In this case, participants rated the expressions as more intense in female compared with male faces (*p* < .001, neutral faces *p* = .666). This effect was not different for judgments of happiness and trustworthiness. In contrast, the sex of the presented face influenced happiness and trustworthiness judgments differentially for male and female participants and this was not influenced by the emotional expression (sex * sex-stim * task: *F*(1,134) = 6.59, *p* = .014, _*p*_*η*^*2*^ = .047, see Table 3, Fig 2). To further explore this effect, we calculated separate analyses for happiness and trustworthiness ratings. For happiness ratings, male and female participants did not differ depending on the sex of the presented face (Mann-Whitney U for the difference between happiness ratings of male and female faces compared for men and women: *Z* = -3.02, *p* = .637). For trustworthiness ratings, in contrast, men and women differed depending on the sex of the displayed character (Mann-Whitney U for the difference between trustworthiness ratings of male and female faces compared for men and women: *Z* = -3.30, *p* < .001). Women rated female faces as more trustworthy than male faces (Wilcoxon signed-rank test: women *Z* = -6.16, *p* < .001, men *Z* = -1.14, *p* = .253). Please note that the interpretability of the other significant effects was restricted due to the higher-order interaction effects.

**Table 3:** Results of the 2x2x2x2 robust rank-aligned ANOVA for social judgments depending on the participants’ sex (sex), the sex of the presented face stimulus (sex-stim), the emotional expression (emotion) and the type of social judgment (task).

	F_(1,134)_	p	_p_η^2^
**sex**	0.13	.717	.001
sex-stim	42.81	< .001	.242
sex*sex-stim	6.59	.014	.047
emotion	964.13	< .001	.878
sex * emotion	0.92	.339	.007
task	167.50	< .001	.556
sex * task	1.60	.208	.012
sex-stim*emotion	121.42	< .001	.475
sex * sex-stim * emotion	0.95	.331	.007
sex-stim*task	10.14	.002	.070
sex*sex-stim*task	7.95	.006	.056
emotion*task	216.06	< .001	.617
sex * emotion * task	2.09	.151	.015
sex-stim * emotion * task	0.96	.330	.007
sex * sex-stim * emotion * task	0.37	.544	.003

Please note: Statistically significant effects are highlighted in red.

### Covariation of social judgments

#### Between-stimuli correlation

Correlation analyses revealed a strong positive correlation between happiness and trustworthiness judgments in both neutral and calm faces when investigating the relation across different facial stimuli based on rating scores averaged across participants (neutral: *r*_*s*_ = .870, calm: *r*_*s*_ = .772, both *p* < .001, see Fig 2a).

#### Between-subject correlation

Correlation analyses revealed positive correlations between happiness and trustworthiness judgments in both neutral and calm faces when investigating the relation across different participants based on rating scores averaged across facial stimuli (neutral: *r*_*s*_ = .228, p = .008, calm: *r*_*s*_ = .417, p < .001, see Fig 2b).

#### Within-subject correlation

Correlation analyses revealed positive correlations between happiness and trustworthiness judgments in both neutral and calm faces when averaging the within-subject correlation coefficients (neutral: Fisher’s *Z* = .306, r = .297, calm: Fisher’s *Z* = .390, *r* = .371, both *p* < .001, see Fig 2c). Distributions of the within-subject correlation coefficients between happiness and trustworthiness judgments differed for neutral and calm faces (Kolmogorov-Smirnov *d* = 0.21, *p* < .001). Please note that the distribution of within-subject correlations between happiness and trustworthiness ratings reveal a high variability in the association between both social judgments between participants (see Fig 2c).

**Fig 3:**
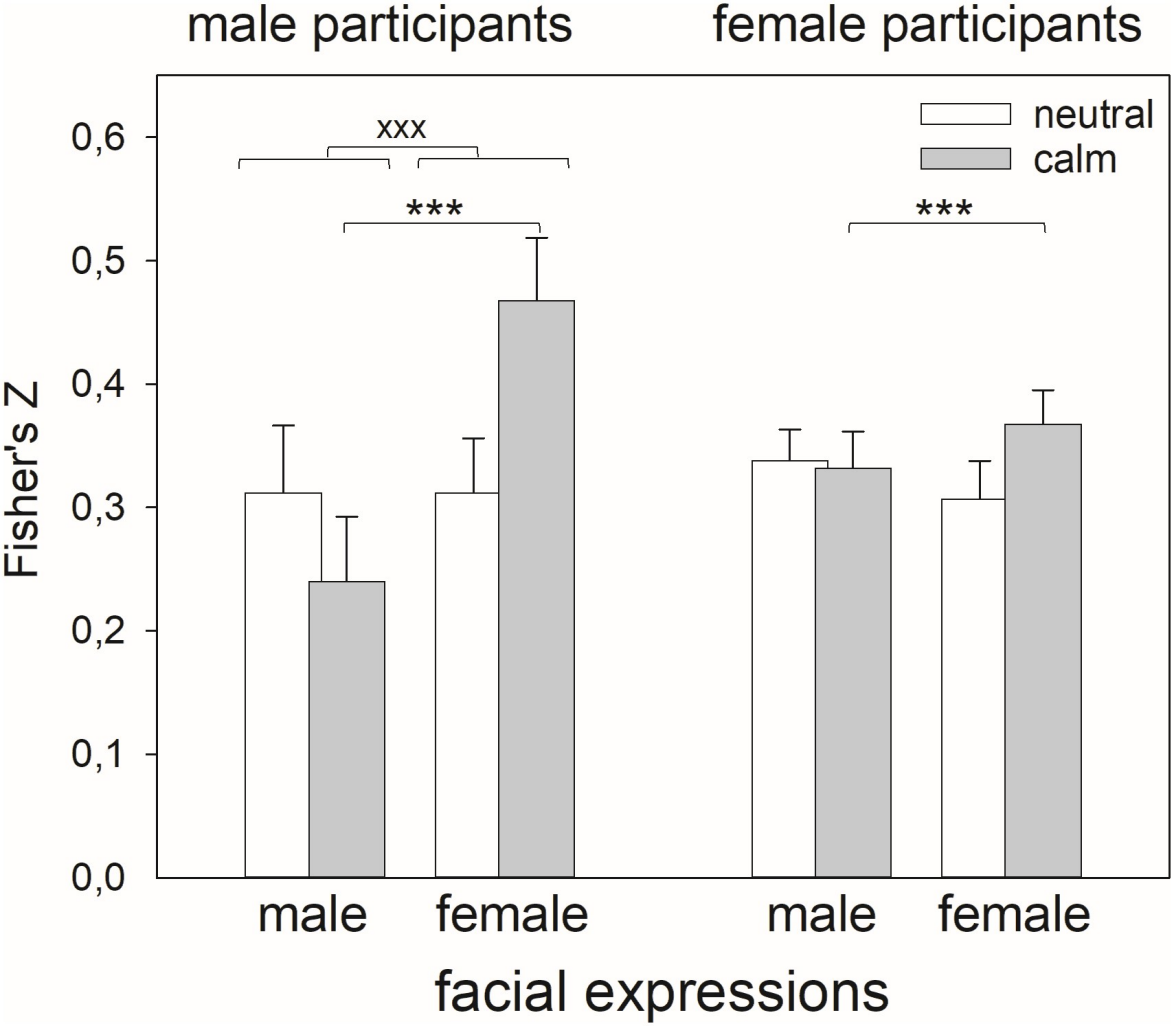
Mean and standard error for Fisher’s z-scores estimating the within-subject relationship between happiness and trustworthiness judgments in neutral and calm female and male facial expressions separated for male and female participants please note: *** indicates p < .001 for the interaction effects of ‘sex * sex-stim’, xxx indicates significant pairwise comparisons with p < .001 for the interaction effect ‘sex-stim * emotion’.

We analysed the influence of the participants’ sex, the sex of the presented faces and the emotional expression on within-subject relations between happiness and trustworthiness judgments with a 2x2x2 rm-ANOVA. The dependent variable was the correlation coefficient transformed to Fisher’s z-score. The relation between both types of judgments differed for male and females faces dependent on the sex of the participants (*F*(1,134) = 5.13, *p* = .025, Table 4): While the sex of the judged faces influenced the correlation in men (*p* = .012), there was no significant difference between male and female faces in women (*p* = .932). Moreover, the sex of the judged faces influenced the correlation depending on the emotional expression (*F*(1,134) = 11.69, *p* = .001): for female faces, there was a stronger correlation between both judgments than for male faces in case of a calm expression (*p* < .001), while there was no difference for neutral expressions (*p* = .641). While the mean scores displayed in Fig 3 suggest that the male participants drive this effect, statistical analyses did not confirm this revealing an only marginally significant effect for the 3-way interaction (*F*(1,134) = 3.49, *p* = .064).

**Table 4:** Results of the 2x2x2 rm-ANOVA for within-subject covariation between happiness and trustworthiness ratings depending on the participants’ sex (sex), the sex of the presented face stimulus (sex-stim) and the emotional expression (emotion).

	F_(1,134)_	p	_p_η^2^
**sex**	0.01	.925	< .001
sex-stim	5.13	.025	.037
sex*sex-stim	4.76	.031	.034
**emotion**	1.21	.272	.009
sex * emotion	0.06	.813	< .001
sex-stim*emotion	11.69	.001	.080
sex * sex-stim * emotion	3.49	.064	.025

Please note: Statistically significant effects are highlighted in red.

### Factor influencing between-subject variability in the within-subject relation of both social judgments

To explore which factors may influence the variability in the interplay of the two social judgments between participants, we calculated correlations of within-subject Fisher’s z-scores with current mood, psychological distress and justice sensitivity. Since the preceding analyses suggest that these factors may differ between men and women, we calculated the analyses only for female participants due to the small percentage of men in the present study. We calculated Fisher’s z-scores used in these exploratory analyses across all experimental condition. Analyses revealed neither a correlation with SAM-valence and SAM-arousal nor with BSI-scores (all *p*>.270). However, the higher participants’ concerns about justice from the perspective of a victim, the higher was the association between both social judgments (*r*_*s*_ = .230, *p* = .019). There was no association with other-oriented concerns about justice, i.e. the perspectives of an observer, a perpetrator or a beneficiary (all *p*>.580).

## Discussion

In the present study, we investigated the interplay between happiness and trustworthiness judgments based on facial appearance. We were particularly interested in whether the high co-variation between both social judgments described in the literature can also be observed between and within participants and whether this relation is influenced by in-group-biases, e.g. the congruency between the sex of the judging and judged individual. Our results replicate previous findings of a positive association between happiness and trustworthiness judgments. We extend our understanding of the link between both judgments by uncovering that between-subject variability in the within-subject association between both types of social judgments is high and influenced by sex and the availability and appraisal of positive emotional social cues.

### Effects of the emotional expression

A positive emotional expression resulted not only in higher happiness ratings, but also in higher trustworthiness ratings when compared with neutral expressions displayed by the same character. While participants assigned to a higher extent trustworthiness to facial expressions than happiness, trustworthiness ratings differed from happiness ratings more strongly in neutral facial expressions. This finding might be explained by a reduced availability of positive social cues that might be interpreted as informative about the willingness of a person to affiliate, suggesting trustworthiness. We found these effects in a rather small sample of 30 participants recruited in Germany and replicated them in a larger sample recruited in the Netherlands.

In line with previous studies, we found a high correlation between happiness and trustworthiness ratings. However, our data suggest that the strength of this relation strongly depends on the chosen methodological approach. When taking the same methodological approach as previously described in the literature, i.e. calculating the correlation between the types of social judgments across facial stimuli based on ratings averaged across participants, we find the same high correlation coefficients as reported in previous studies. This finding is in line, for example, with principal component analyses in neutral natural and computer-generated faces investigating underlying dimensions of trait judgments: they revealed very high positive factor loadings (all equal or higher than 0.90) of trustworthiness judgments on the valence dimension [16].

However, when looking on this relation based on average ratings across facial stimuli, this association is less close. Within-subject analyses even revealed that the strength to which an emotional expression relates to trustworthiness judgments is highly variable between subjects. This implies that people differ in the extent to which both types of social judgments indeed rely on the same mechanism and modulating factors. So far, experimental studies on the involved mechanisms are sparse. A recent study by Calvo and colleagues [33] suggests that both judgments rely on overlapping but also distinct attentional mechanisms. While happiness judgments were linked to a higher fixation density for the mouth region of a face, trustworthiness judgments were related with a higher fixation density in the eye region. Moreover, fixation durations were higher during trustworthiness compared with happiness evaluations indicating increased attentional demands or processing.

### Effects of sex

The sex of the participants resulted in difference in trustworthiness ratings depending on the sex of the displayed facial stimulus. Female faces were rated more trustworthy, but this effect was only true for female participants. This finding is in line with previous studies supporting that women experience same-sex individuals as more trustworthy [13]. Our findings extend previous studies by confirming that this is true particularly for trustworthiness judgments since no comparable effect was found for happiness ratings. Contrarily, male participants did not show a comparable same-sex advantage for attributing trustworthiness. However, beyond this in-group bias in women, our data also showed that all participants evaluated the female facial stimuli as more positive when they displayed a calm expression, that is, they assessed both the happiness as well as the trustworthiness as more intense in female compared with male faces. These findings point to a potential issue in studying the influence of sex of facial stimuli on different social judgments: stimulus material might be biased toward a more intense expression of positive valence in female faces constituting a confounding factor when analysing sex differences in trustworthiness assessments. This emphasizes the need to control stimulus material for differences between sexes in the intensity of an emotional expression when investigating the effects of sex on trustworthiness.

### Association with individual features of the participants

Exploratory analyses revealed no relation of within-subject relations between social judgments to current mood or psychological and somatic wellbeing. This is in line with a study by Dong and colleagues that showed the relation of trustworthiness and happiness judgments to be independent of the perceiver’s mood [34]. The only link our exploratory analyses revealed was that people with higher concerns about justice showed a stronger association between happiness and trustworthiness ratings. This was true only for concerns about justice from the perspective of a victim, but not for other-oriented types of justice sensitivity, that is the perspective of an observer, a perpetrator or beneficiary. A previous study found that people high in victim sensitivity rated neutral and hostile faces as more untrustworthy than people low in victim sensitivity [35]. In contrast, there was similar association for friendly faces. In line, we found no correlation with overall trust ratings in our sample. However, our data revealed that participants high in victim-sensitivity rated happiness as more intense. One may speculate that victim sensitive people differ in the appraisal of positive social cues and use these social cues in a higher extent to determine trustworthiness. However, since we did not correct these exploratory analyses for multiple testing, they have to be interpreted with care and be replicated in future studies.

### Limitations

Several limitations of the presented study have to be mentioned. First, women were overrepresented in both samples, which restricts the interpretability of findings for men and warrants caution to the interpretation of the sex analyses due to the unbalanced design. Thus, further studies are needed that replicate our findings in a balanced design with carefully matched samples of men and women. Both samples included participants of other nationalities than German or Dutch. This may be considered as limitation of the present study since cultural differences may affect social cognition such as emotion recognition in facial or vocal expressions [36–38]. The question whether and how culture may affect the reported findings has to be investigated in future studies taking differences for example in social trust in different countries into account [39]. Moreover, the experimental task was done as part of an online survey in contrast to a laboratory setting that allows for more controlled experimental conditions. Future studies have to replicate the current findings and use additional dependent variables such as reaction times and brain imaging to contribute to a deeper understanding of differences in the involved cognitive processes. Additionally, a link to social functioning and experimental paradigms that assess behavioural correlates of trust instead of explicit trustworthiness appraisals seem to be important extensions of the current study. Beyond this, the interplay between both domains of processing has to be investigated in different contexts, since context and the related specific goals of the appraisal processes have been shown as an important modulators of trustworthiness judgments [25]. Finally, we focused on the relationship between trustworthiness and happiness. Whether a comparable interplay exist between the emotional processing of negative social cues such as anger and untrustworthiness has to be investigated in future studies. Colonello and colleagues [40] could show that particularly the untrustworthiness of facial stimuli affect the recognition of negative emotions, while there was no advantage effect in happiness recognition for trustworthy faces.

### Implications

Our study has some implications for studies on trustworthiness in general as well as in clinical populations. Our findings emphasize the need to control for the intensity of emotional expression when interested in differences in trustworthiness of male and female faces, since females faces have been assessed as both happier as well as more trustworthy. Moreover, effects between the processing of male and female faces may differ in the different domains of processing between men and women, which would restrict the generalizability of findings when the different sexes are not taken into account. Issues with trust are a central symptom domain in many mental disorders such as psychotic disorders or personality disorders like Borderline Personality Disorder. However, in most of these disorders studies also revealed impairments of emotion recognition abilities (e.g. [41]). Our findings emphasize the need for studies in clinical samples to study trust in the context of emotion processing. If impairments of trust can be traced down at least partially to impairments of emotion recognition, interventions on trust might be inefficient without the simultaneous training of the recognition of emotions and the patients’ confidence in being able to assess these [42]. This view is supported by several studies revealing that a variation in the emotional expression does influence cooperative behaviour to a smaller extent in clinical samples compared to healthy control subjects (e.g. [43]). In this context, it seems important to mention that clinical studies even used trustworthiness appraisal tasks to measure implicit emotion recognition [44].

### Summary

In sum, the present study confirms the relevance of positive social cues provided by a facial expression for trustworthiness judgments. It extends our understanding of the interplay between both social judgments by showing that the closeness of the relation between both varies profoundly between individuals. We uncovered first factors that contribute to this inter-subject variability, since both the sex of the judging and the judged individual as well as concerns about justice from the perspective of a victim influences this relationship. However, further studies have to elucidate the different mechanisms of both judgments as well as the determinants of their interplay.

## Supporting information

S1 Dataset (SAV)
